# Health-Related Quality of Life Among High-Risk Pregnant Women Hospitalized in a Maternal-Fetal Intensive Care Unit: A Structural Equation Modeling Analysis

**DOI:** 10.3390/healthcare13192534

**Published:** 2025-10-07

**Authors:** Hyuna Seol, Jina Oh, Mihae Im

**Affiliations:** 1 Nursing Department, Inje Paik Hospital, Busan 47392, Republic of Korea; 033530@paik.ac.kr; 2 College of Nursing, Inje University, Busan 47392, Republic of Korea; 3 Department of Nursing, Daegu Haany University, Gyeongsan 38610, Republic of Korea; mihae1219@gmail.com

**Keywords:** attachment, depression, health-related quality of life, high-risk pregnancy, self-care

## Abstract

**Objectives**: The aim of this study was to establish a structural model that could explain and predict factors influencing health-related quality of life (HRQoL) in high-risk pregnant women. **Methods**: This study adopted a structural equation model based on the HRQoL model. Data were collected from 200 high-risk pregnant women hospitalized in a maternal-fetal intensive care unit. Structured questionnaires measured antenatal depression, marital intimacy, fetal attachment, self-care behavior, perceived health status, and HRQoL. All data were collected simultaneously during a single point in participants’ hospitalization. **Results**: Among the factors, antenatal depression showed the greatest influence on HRQoL (β = −0.27, *p* < 0.001), followed by marital intimacy (β = 0.26, *p* < 0.001), fetal attachment (β = 0.25, *p* < 0.001), perceived health status (β = 0.14, *p* = 0.005), and self-care behavior (β = 0.12, *p* = 0.037), with an explanatory power of 73.4%. **Conclusions**: To improve the HRQoL of high-risk pregnant women, psychological nursing intervention strategies are needed to reduce antenatal depression. It is necessary to provide education that encourages self-care behavior. Furthermore, the findings indicate that strategic nursing interventions are necessary to enhance marital intimacy, fetal attachment, and perceived health status. As a theoretical basis, this study will contribute to the preparation of basic data that will improve the HRQoL of pregnant women at high risk.

## 1. Introduction

As the number of women of advanced maternal age increases, the incidence of complications that threaten the course of pregnancy also rises, leading to a greater number of high-risk pregnancies [1,2]. High-risk pregnant women often suffer from physical symptoms such as premature labor, nausea and vomiting due to uterine inotropic drug administration, bleeding associated with placenta previa, and headache and edema resulting from gestational hypertension [3]. These complications increase the likelihood of maternal mortality and the birth of high-risk newborns [3,4]. Consequently, high-risk pregnant women experience physical and psychological instability during pregnancy [3,4], which can significantly affect their health-related quality of life (HRQoL) [5].

Ferrans et al. [6] presented the HRQoL model by identifying the personal and environmental characteristics influencing biological function, as well as their direct and indirect effects. Recent research on the HRQoL of people with diseases has underscored the important effects of individuals’ positive health behaviors on biological function [7,8]. Accordingly, in an era of low birth rates in which the health of both mothers and infants is emphasized, the model can be applied to high-risk pregnant women as a theoretical foundation to investigate factors affecting HRQoL.

**Figure 1:**
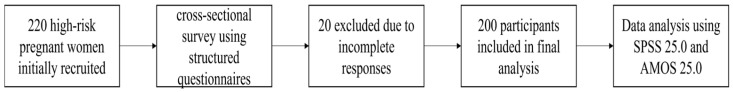
Flow chart of participant recruitment and data analysis procedures.

In this study, a hypothetical model was constructed based on the HRQoL model and a literature review of Ferrans et al. [6], who revised the conceptual model of Wilson and Cleary [9]. Exogenous variables included personal characteristics (age, gestational week, and planned pregnancy) and environmental characteristics (marital intimacy and social support) as factors influencing HRQoL [10]. Endogenous variables included biological function (number of high-risk gestational diseases) due to complications of high-risk pregnancies that occur with other diseases as underlying variables, psychological factors affecting the health outcomes of mothers and babies include symptom status (antenatal depression), the role functions of high-risk pregnant women and optimizing their current functions focused on functional status (fetal attachment and self-care behavior), general health perception (perceived health status), and HRQoL (Figure 1) [7,11].

The present study aimed to develop and test a structural equation model that could explain and predict factors influencing HRQoL in high-risk pregnant women hospitalized in the maternal-fetal intensive care unit (MFICU) and to investigate the direct or indirect effects of related variables on HRQoL in high-risk pregnant women. The specific objectives of this study were as follows: (i) to construct a hypothetical model explaining the HRQoL of high-risk pregnant women; (ii) to test the goodness of fit between the hypothetical model and actual data; (iii) to present a model that could explain and predict the HRQoL in high-risk pregnant women; and (iv) to assess the direct, indirect, and total effects of variables influencing the HRQoL of high-risk pregnant women and to confirm the causal relationship between variables.

## 2. Materials and Methods

### 2.1. Study Design

This study employed structural equation modeling. A hypothetical model of HRQoL and its influencing factors in high-risk pregnant women was developed by conceptualizing the theoretical framework proposed by Ferrans et al. [6] and reviewing the relevant literature (Figure 1).

### 2.2. Participants

This study targeted high-risk pregnant women hospitalized in the MFICU of Paik University Hospitals, Busan, as participants. Given that more than 220 participants were suitable for the maximum likelihood method of structural equations [12], 220 participants were sampled, with a dropout rate of 10%. After excluding 20 insufficient copies, data of 200 participants were used for the final analysis (Figure 1).

In this study, only high-risk pregnant women who were hospitalized in the MFICU due to clinical complications were included. Hospitalization criteria followed the 2016 guidelines of the Korean Society of Obstetrics and Gynecology and included conditions such as preterm labor, placenta previa, premature rupture of membranes, gestational hypertension, severe ultrasonographic anomalies, and other maternal or fetal complications requiring inpatient monitoring and treatment.

The inclusion criteria were as follows: (i) pregnant women who were diagnosed with a high-risk pregnancy and admitted to the MFICU for the first time; (ii) high-risk pregnant women with a gestational age between 20 and 34 weeks; and (iii) high-risk pregnant women who understood the purpose of this study and voluntarily agreed to participate. The exclusion criteria were as follows: high-risk pregnant women who had psychiatric diseases (e.g., cognitive dysfunction and depression) or were readmitted to the MFICU.

### 2.3. Measurements

#### 2.3.1. Demographic Information

The general characteristics of high-risk pregnant women, including their age, obstetric history, religion, occupation, education level, average monthly income, planned pregnancy, and family structure, were investigated. Disease-related characteristics, such as high-risk pregnancy diagnoses, number of high-risk pregnancy diseases, number of weeks of pregnancy, and infertility treatment, were also examined.

#### 2.3.2. Social Support

Social support was evaluated using the Multidimensional Scale of Perceived Social Support, which was developed by Zimet et al. [13] and translated by Shin and Lee [14]. The participants were asked to respond using a five-point Likert scale with 12 questions. The Cronbach’s alpha of this scale was 0.83 [13] at the time of development, 0.89 in a previous study [14], and 0.91 in this study.

#### 2.3.3. Marital Intimacy

Marital intimacy was assessed using the scale developed by Lee [15]. This scale employed a five-point Likert scale ranging from 1 (strongly disagree) to 5 (strongly agree), with 15 questions. The total score ranged from a minimum of 15 to a maximum of 75, with higher total scores indicating higher marital intimacy. The Cronbach’s alpha of this scale was 0.90 in Lee’s study [15] and 0.88 in this study.

#### 2.3.4. Antenatal Depression

Antenatal depression was assessed using the Edinburgh Postnatal Depression Scale originally developed by Cox et al. [16] and translated by Han et al. [17]. This scale was developed to screen for postpartum depression, but its validity for use throughout pregnancy has been confirmed [18]. This scale employed a four-point Likert scale ranging from 0 to 3, with 10 questions (0–30 points). The Cronbach’s alpha of this scale was 0.87 at the time of development [16], 0.85 in a previous study [17], and 0.84 in this study.

#### 2.3.5. Self-Care Behavior

Self-care behavior was measured using the Prenatal Care Scale originally developed by Lee [19] and modified by Park [20]. This scale comprised 25 questions scored on a five-point Likert scale related to frequency. The Cronbach’s alpha of this scale was 0.81 at the time of development [19], 0.82 in a previous study [20], and 0.80 in this study.

#### 2.3.6. Perceived Health Condition

Perceived health condition was examined using the Perceived Health Status Scale originally developed by Speak et al. [21] and translated by Whang [22]. This scale comprised three questions with a five-point Likert scale. The Cronbach’s alpha of this scale was 0.85 at the time of development [21], 0.85 in a previous study [22], and 0.81 in this study.

#### 2.3.7. Fetal Attachment

Fetal attachment was measured using the Maternal-Fetal Attachment Scale developed by Cranley [23] and translated by Kim [24]. The participants were asked to respond using a five-point Likert scale with 24 questions. The Cronbach’s alpha of this scale was 0.85 [23] at the time of development, 0.89 in a previous study [24], and 0.91 in this study.

#### 2.3.8. HRQoL

HRQOL was evaluated using the Korean version of the World Health Organization Quality of Life-BREF (WHOQOL-BREF) [25]. This scale consisted of 26 questions with a five-point Likert scale. The Cronbach’s alpha for the Korean version of the WHOQOL-BREF was 0.89 in a previous study [25] and 0.92 in this study.

### 2.4. Data Collection

The Institutional Review Board at Inje University approved this study (approval no. 2020-04-021-002). After seeking cooperation from the heads Paik University Hospitals located in Busan in Korea, data were collected between 12 June 2020, and 28 February 2021, from high-risk pregnant women hospitalized in the MFICU. The researchers directly explained the purpose of this study to these high-risk pregnant women and obtained voluntary consent from those who met the selection criteria. The completed questionnaires were immediately collected in a sealed document envelope; these questionnaires could be completed within approximately 10–15 min. All study variables were measured simultaneously at a single point in time during participants’ hospitalization. Goods for pregnant women were provided as small gifts to all participants.

### 2.5. Data Analysis

Data were analyzed using IBM SPSS version 25.0 and AMOS version 25.0 (IBM Corp., Armonk, NY, USA). Skewness and kurtosis were estimated to confirm the normal distribution of variables. Multicollinearity between the measured variables was analyzed using the tolerance and variance inflation factor. The validity of the model was confirmed through a confirmatory factor analysis. The structural equation model was tested by conducting a covariate structural analysis with the maximum likelihood method, assuming multivariate normality. The suitability of this research model for the data was evaluated by performing a goodness-of-fit test using χ^2^, χ^2^/df, standardized root mean square residual (SRMR), root mean square error of approximation (RMSEA), Tucker–Lewis index (TLI), and comparative fit index (CFI). The significance of the path of this research model was confirmed by the standardized coefficient, standard error, critical ratio, and *p*-value. The explanatory power of endogenous variables was confirmed by squared multiple correlation (SMC). Modification of the model was guided by both theoretical justification and statistical criteria (modification indices), with caution to minimize overfitting.

## 3. Results

### 3.1. Demographic Information of Participants

The average age of the participants was 34.2 years, and 97 (48.5%) had a job (Table 1). Furthermore, 77 (38.5%) participants had at least one high-risk pregnancy disease. Among the participants with high-risk pregnancy diagnoses, pregnant women over 35 years of age comprised the majority (45%, n = 90), followed by those with preterm labor (35.5%, n = 71).

### 3.2. Descriptive Statistics, Multicollinearity, and Normality of Measured Variables

The skewness and kurtosis values of the total scores ranged from −1.27 to 0.00 and from −0.36 to 1.59, respectively (Table 2). The absolute skewness value was below 3.0, whereas the kurtosis value did not exceed 10.0, confirming that the normality assumption was met. When the multicollinearity between the study variables was assessed, the variance inflation factor and tolerance values were below 10 (1.11–3.35) and over 0.1 (0.30–0.90), indicating that there was no problem with multicollinearity.

### 3.3. Test of the Hypothetical Model

#### 3.3.1. Reliability and Validity of Measures

Standardized factor loading was checked as a method for verifying the degree of consistency among variables; the results indicated standardized factor loading values of 0.64–0.81 for social support, 0.65–0.85 for marital intimacy, 0.65–0.82 for fetal attachment, and 0.68–0.87 for HRQoL, all of which were 0.50 or higher, securing convergent validity. Furthermore, construct reliability, which indicated the internal consistency between latent variables, was confirmed to be 0.91–0.95 (all above 0.79), and the average variance was 0.78–0.83 (all above 0.50), thereby confirming convergent validity. The correlation coefficient between latent variables was 0.37–0.66, which was below 0.90; additionally, the squared value of all correlation coefficients was 0.14–0.42, which confirmed discriminant validity because it did not exceed the average variance.

#### 3.3.2. Goodness of Fit of the Model

The hypothetical model structure was examined through maximum likelihood estimation (Table 3). The goodness of fit was χ^2^ = 456.81 (*p* < 0.001), χ^2^/df = 2.47, TLI = 0.83, CFI = 0.84, SRMR = 0.02, and RMSEA = 0.08. Consequently, the model was modified based on the modification index (MI). The paths from self-care behavior to fetal attachment (MI = 12.254) and from social support to marital intimacy (MI = 10.992) were added. Furthermore, Correlation between fetal attachment differentiation and measurement errors in role acquisition (MI = 14.020), Correlation between the special support of social support and the measurement error of emotional in marital intimacy (MI = 5.489), and Correlation of measurement error in emotional and sexual in marital intimacy (MI = 4.304) were added. The goodness of fit of the modified model was χ^2^ = 395.70 (df ≤ 3, *p* < 0.001), χ^2^/df = 2.2, TLI = 0.86, CFI = 0.90, SRMR = 0.02, and RMSEA = 0.07, reaching the recommended level. Therefore, the modified model with improved goodness of fit, as compared with the hypothetical model, was adopted.

#### 3.3.3. Path Estimates of the Modified Model

**Figure 2:**
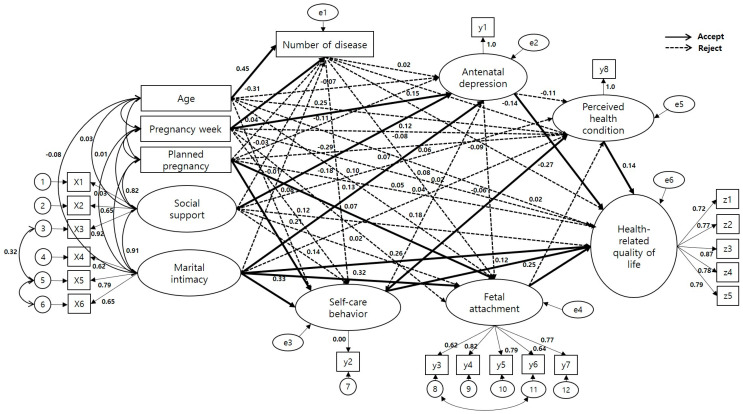
Path diagram for the modified model.

The parameter estimates and statistical significance of the modified model were verified; the results indicated that 15 out of 44 paths were significant, whereas 29 paths were not significant (Table 4, Figure 2).

First, age (β = 0.44, *p* < 0.001) and gestational weeks (β = −0.31, *p* < 0.001) showed a significant effect on the number of high-risk gestational diseases. The SMC was 27.8%, with age being the most influential factor.

Second, gestational weeks (β = −0.25, *p* < 0.001), social support (β = −0.29, *p* = 0.001), and marital intimacy (β = −0.18, *p* = 0.044) were found to have a significant effect on antenatal depression. The SMC was 26.6%, with social support being the most influential factor.

Third, planned pregnancy (β = 0.20, *p* < 0.001) and marital intimacy (β = 0.33, *p* < 0.001) had a significant effect on self-care behavior. The SMC, which indicated the explanatory power of planned pregnancy and marital intimacy for self-care behavior, was 29.8%, with marital intimacy being the most influential factor.

Fourth, planned pregnancy (β = 0.20, *p* < 0.001) and marital intimacy (β = 0.32, *p* = 0.002) showed a significant effect on fetal attachment. The SMC was 39.3%, with marital intimacy being the most influential factor.

Fifth, self-care behavior (β = 0.17, *p* = 0.035) had a significant effect on perceived health status. The SMC, which indicated the explanatory power of self-nursing behavior for perceived health status, was 16.3%.

Sixth, marital intimacy (β = 0.26, *p* < 0.001), antenatal depression (β = −0.27, *p* < 0.001), self-care behavior (β = 0.12, *p* = 0.037), fetal attachment (β = 0.25, *p* < 0.001), and perceived health status (β = 0.14, *p* = 0.005) had a significant effect on HRQoL. The SMC was 73.4%, with antenatal depression being the most influential factor.

#### 3.3.4. Direct, Indirect, and Total Effects of the Modified Model

Among the 44 paths of the model, 14 paths exhibited significant direct effects, nine paths had significant indirect effects, and 18 paths showed significant total effects with direct and indirect effects (Table 5).

Marital intimacy (β = 0.26, *p* = 0.001), antenatal depression (β = −0.27, *p* = 0.001), self-care behavior (β = 0.12, *p* = 0.037), fetal attachment (β = 0.25, *p* = 0.001), and perceived health status (β = 0.14, *p* = 0.005) were identified as significant variables that directly affected HRQoL.

Gestational age (β = 0.19, *p* = 0.008), planned pregnancy (β = 0.06, *p* = 0.010), social support (β = 0.14, *p* = 0.017), marital intimacy (β = 0.22, *p* = 0.006), and self-care behavior (β = 0.11, *p* = 0.007) were found to be significant variables that indirectly affected HRQoL.

Significant variables that showed a total effect on HRQoL were as follows: gestational age (β = 0.24, *p* = 0.021), social support (β = 0.27, *p* = 0.044), marital intimacy (β = 0.48, *p* = 0.005), number of high-risk pregnancy diseases (β = −0.14, *p* = 0.022), antenatal depression (β = −0.31, *p* = 0.007), self-care behavior (β = 0.23, *p* = 0. 011), fetal attachment (β = 0.25, *p* = 0.012), and perceived health status (β = 0.14, *p* = 0.005).

## 4. Discussion

A structural model was developed in this study that explains the HRQoL of pregnant women with high risk and validates the causal relationship between factors that impact HRQoL. Accordingly, we would like to discuss the suitability of the HRQoL model constructed during this study as well as influencing factors of the HRQoL of high-risk pregnant women revealed as a result of the verification.

Although the model’s fit was improved through the removal of non-significant paths, only 15 out of 44 paths were initially significant. This raises the potential risk of overfitting due to excessive model modification based solely on statistical criteria. Overfitting can result in limited generalizability and compromised interpretability of the model. Future studies should aim to replicate the model using larger, independent samples, and retain only theoretically and statistically robust paths to enhance external validity and ensure more stable results.

The hypothesis model was found to be suitable with regard to statistics, but some statistics confirming the fitness did not meet the standard value, thus the fitness was somewhat lost. In this study, there were a total of 44 hypotheses, and it is believed that the suitability of the model was lowered because of the numerous correlations between the exogenous variables. Therefore, as a result of reverification by modifying the model, the modified model in the present study explained 73.4% of HRQoL in high-risk pregnant women. A previous study on pregnant women [26] reported an explanatory power of 51% for HRQoL due to spousal psychological violence, social support, and prenatal depression. Kim [27] reported an explanatory power of 49% for HRQoL due to anxiety and social support in pregnant women; in comparison, the explanatory power in the present study was relatively high.

In this study, the average HRQoL score was 71 points out of 100, which is lower than the HRQoL scores of elderly pregnant women (85.8 points) and normal pregnant women (92.6 points) measured using the WHOQOL-BREF [26,28]. This suggests that high-risk pregnant women evaluate their HRQoL as lower because of physical pain and psychological anxiety caused by hospitalization.

Marital intimacy was confirmed to have the greatest impact on the HRQoL of high-risk pregnant women and to have a direct effect on antenatal depression (β = −0.18, *p* = 0.044). Previous studies showed that the level of prenatal depression was lower when the relationship with one’s spouse was amicable [29,30] and that the higher the marital intimacy of healthy pregnant women, the higher their life satisfaction [31]. Jung et al. [32] suggested that aroma hand massages could reduce pregnant women’s stress and improve their marital relationships. It is important to establish social systems that provide spousal support during pregnancy to enhance the QoL and health of high-risk pregnant women. Moreover, it may be advisable to introduce active nursing intervention programs such as massage classes with spouses for high-risk pregnant women in hospitals.

Gestational age (β = −0.26, *p* = 0.011) and social support (β = −0.29, *p* = 0.010) were found to have a direct effect on HRQoL through antenatal depression. In other words, the lower the gestational age and social support, the higher the risk of antenatal depression, which reduces the HRQoL of high-risk pregnant women. Previous studies also confirmed that social support affected antenatal depression in pregnant women in early labor and high-risk pregnant women [33,34]. Therefore, when pregnant women diagnosed with high-risk pregnancy are hospitalized, nursing plans and interventions are required to prevent prenatal depression by fully recognizing the possibility of prenatal depression in advance.

Social support was found to be directly effective in prenatal depression; the higher the social support, the lower the prenatal depression, thereby improving HRQoL. This could mean that separation from family and other people around them due to hospitalization could affect the psychological and emotional states of high-risk pregnant women. This suggests that social support systems such as family, friends, neighbors, and medical personnel are needed, especially for hospitalized women with high-risk pregnancy. Previous studies have consistently reported that appropriate social support during pregnancy improves HRQoL [35,36]. However, Park [37] reported conflicting results, showing that family support, among the different types of social support, did not affect the HRQoL of pregnant women. Therefore, repeated research on factors influencing the social support of pregnant women is necessary.

In this study, fetal attachment had a significant impact on the HRQoL of high-risk pregnant women, whereas planned pregnancy (β = 0.07, *p* = 0.005) and marital intimacy (β = 0.44, *p* = 0.007) had indirect effects on HRQoL through fetal attachment. It is thought that planned pregnancy, which is a health behavior involving the desire to become pregnant, and the emotional intimacy of a spouse could affect the HRQoL of high-risk pregnant women by increasing fetal attachment to form a positive relationship between the pregnant woman and the fetus. This is supported by previous research [26,38] in which fetal attachment behavior is higher in planned pregnancies. In addition, the higher the spousal support and marital intimacy, the higher the fetal attachment in normal or elderly pregnant women [33,38]. Despite prior research on fetal attachment and HRQoL among high-risk pregnant women, there is still a need for ongoing research to clarify these issues.

Gestational age significantly affected the HRQoL of high-risk pregnant women. Gestational age (β = −0.31, *p* < 0.001) directly affected the number of high-risk pregnancy diseases; thus, the lower the gestational age, the higher the number of high-risk pregnancy diseases. Gestational age (β = −0.25, *p* < 0.001) directly affected antenatal depression, with the lower the gestational age, the higher the prenatal depression rate. This may be because gestational age is an important obstetric factor that determines the perinatal prognosis. In addition, according to a previous study [39], as the gestational age progresses, HRQoL increases due to relief from the continuation of pregnancy and attachment to the fetus. An indirect effect of gestational age on fetal attachment was observed in this study, which supports the finding that fetal attachment increases as pregnancy progresses.

Self-care behavior was found to have a significant impact on the HRQoL of high-risk pregnant women. Additionally, gestational age (β = 0.16, *p* = 0.011), planned pregnancy (β = 0.20, *p* = 0.013), and marital intimacy (β = 0.34, *p* = 0.008) were found to have a direct impact on HRQoL through self-care behavior. Previous research has indicated that pregnancy planning and prenatal counseling have a significant impact on self-care behavior [40]. In addition, the results of this study were supported by the finding that the active roles of spouses and family members promote self-care behavior in pregnant women with gestational diabetes [41]. Furthermore, self-care behavior has been shown to have a significant impact on self-care among pregnant women with gestational diabetes [42]. Studies of patients with various diseases have reported that self-care behavior affects HRQoL [43]; therefore, it is necessary to develop education that can improve self-care behavior for each disease.

Perceived health status also had a significant impact on the HRQoL of high-risk pregnant women, and gestational age (β = 0.24, *p* = 0.011) indirectly affected HRQoL through perceived health status. The HRQoL of pregnant women with gestational diabetes was affected by their perceived health status [44]. As perinatal prognosis and newborn health status stabilize, individuals become more aware of their own health, and their HRQoL increases. Efforts should be further expended to increase awareness of diagnosed diseases and health status among high-risk pregnant women. Therefore, a systematic approach to health management education should be applied to improve the perceived health status of high-risk pregnant women.

Finally, the number of high-risk pregnancy diseases significantly affected the HRQoL of high-risk pregnant women. Additionally, it was confirmed that age (β = 0.44, *p* < 0.001) and gestational age (β = −0.31, *p* < 0.001) have a direct impact on HRQoL through the number of high-risk pregnancy diseases. This finding supports the literature claiming that the incidence of high-risk pregnancies is also increasing along with a continuous increase in the number of elderly pregnant women [3] and suggests the need for a nursing approach to improve HRQoL in high-risk pregnant women. In a study by Prick et al. [45], pregnant women diagnosed with intrauterine growth retardation and gestational hypertension showed lower HRQoL than those diagnosed with postpartum hemorrhage, which is consistent with the results of this study. Additionally, studies targeting middle-aged women and the elderly have reported that the number of chronic diseases has a significant impact on HRQoL [11]. Therefore, when determining nursing care for high-risk pregnant women, an integrated approach should be taken that considers age, gestational age, and high-risk nursing interventions.

This study has some limitations. First, although the model’s fit improved by removing non-significant paths based on theoretical rationale, approximately 30% of all hypothetical relationships were statistically significant. Additionally, the model was not validated with an independent dataset, and no a priori power analysis was conducted. These factors may raise concerns regarding overfitting and the statistical robustness of the findings. Future research should replicate the model with larger, independent samples and conduct power analyses to ensure stability and external validity. Second, participants were recruited only during the early stage of hospitalization from a single university hospital in Busan, and all data were collected cross-sectionally. These conditions limit the generalizability of the findings and preclude causal inferences. Furthermore, clinical variables such as gestational age and the number of high-risk pregnancy complications were not considered in the analysis. Third, although psychiatric conditions were excluded to improve the reliability of self-reported data, other medical and obstetric complications as well as medication use (e.g., tocolytics, antihypertensives, and corticosteroids) were not controlled for to reflect real-world clinical diversity. However, this heterogeneity and lack of adjustment for medication-related confounders may limit the interpretability of the findings. Future studies should consider stratifying or adjusting for specific clinical and pharmacological conditions. Lastly, a stratified analysis by participant characteristics such as age group, type of obstetric complication, or socioeconomic background was not performed owing to limited sample size. As a subgroup analysis would have reduced statistical power, future studies should use larger and more heterogeneous populations to investigate the differential impacts of these factors on HRQoL.

## 5. Implications for Clinical and Academic Practice

Based on the findings of this study, the following implications can guide both clinical practice and academic nursing education:

⬤ Early assessment of resilience and perceived health status may help identify high-risk pregnant women at greater risk for diminished HRQoL during hospitalization.
⬤ Targeted nursing interventions to enhance individual resilience and perceived family support should be incorporated into maternal care in the MFICU.
⬤ Educational programs for nurses should emphasize psychological and relational aspects of care for high-risk pregnant women.
⬤ Routine use of HRQoL-focused assessment tools can support individualized and patient-centered care planning.
⬤ Research and curricula in academic nursing should integrate the multifactorial nature of HRQoL and structural modeling approaches to improve evidence-based practice.

## 6. Conclusions

Antenatal depression, self-care behavior, marital intimacy, fetal attachment, and perceived health status were found to significantly influence the HRQoL of high-risk pregnant women, accounting for 73.4% of the variance. The results of this study are significant as they confirmed the HRQoL of high-risk pregnant women by focusing on various aspects of women diagnosed with high-risk pregnancies and hospitalized in the MFICU. Additionally, the identification of influencing factors was made significant by factoring fetal attachment, including fetal health, into the functional status. To improve the HRQoL of high-risk pregnant women, strategic nursing interventions are needed to reduce prenatal depression, strengthen self-care behavior, and improve couple intimacy, fetal attachment, and perceived health status. Therefore, this study contributes to the theoretical framework for preparing basic data to improve the HRQoL of high-risk pregnant women.

## Figures and Tables

**Table 1:** Demographic information of the participants. (n = 200).

Characteristics	Categories	N (%) or M ± SD
Age (years)		34.19 ± 4.20
	20~29	28 (14.0)
	30~39	151 (75.5)
	40~49	21 (10.5)
Religion	Yes	60 (30.0)
	No	140 (70.0)
Occupation	Yes	97 (48.5)
	No	103 (51.5)
Education	≤High school	25 (12.5)
	≥College	175 (87.5)
Number of pregnancies		1.16 ± 1.15
	0	69 (34.5)
	1	61 (30.5)
	2	50 (25.0)
	≥3	20 (10.0)
Number of births		0.51 ± 0.73
	0	122 (61.0)
	1	58 (29.0)
	≥2	20 (13.0)
Number of miscarriages	0	127 (63.5)
	1	47 (23.5)
	2	21 (10.5)
	≥3	5 (2.5)
Number of children		0.44 ± 0.63
	0	125 (62.5)
	1	63 (32.5)
	≥2	10 (5.0)
Monthly income		543.45 ± 0.62
(10,000 KRW)	<300	23 (11.5)
	300~500	76 (38.0)
	501~700	60 (30.0)
	>700	41 (20.5)
Family type	Large family	8 (4.0)
	Nuclear family	182 (91.0)
	Others	10 (5.0)
Planned pregnancy	Yes	75 (37.5)
	No	125 (62.5)
High-risk pregnancy	Over 35 years of age	90 (45.0)
diagnosis	Gestational diabetes	17 (8.5)
(multiple responses)	Cervical incompetency	25 (12.5)
	Placenta previa	25 (12.5)
	Oligohydramnios	8 (4.0)
	Intrauterine growth restriction	16 (8.0)
	Multiple pregnancy	27 (13.5)
	Gestational hypertension	14 (7.0)
	Premature rupture of membrane	19 (9.5)
	Labor pain	71 (35.5)
	Ultrasonic abnormality	30 (15.0)
	Mother with a condition that can affect pregnancy	52 (26.0)

KRW = Korean won; M = Mean; SD = Standard deviation.

**Table 2:** Convergent validity of latent variables (n = 200).

Variables		M ± SD	Scale Range	Skewness	Kurtosis	Standardized Factor Loading	SE	CR	AVE
Social support	Family support	4.49 ± 0.56	1–5	−1.27	1.59	0.81		0.91	0.78
	Friends support	3.95 ± 0.82	1–5	−0.57	−0.05	0.64	0.12		
	Others support	4.48 ± 0.58	1–5	−1.13	0.67	0.92	0.08		
Marital intimacy	Cognitive	3.86 ± 0.59	1–5	−0.48	0.61	0.85		0.93	0.83
	Emotional	3.82 ± 0.65	1–5	−0.44	−0.36	0.85	0.08		
	Sexual	3.52 ± 0.65	1–5	0.00	−0.22	0.72	0.08		
Fetal attachment	Fetal division	4.11 ± 0.59	1–5	−0.33	−0.11	0.65		0.94	0.76
	Interaction	4.11 ± 0.62	1–5	−0.58	0.10	0.82	0.13		
	Intent estimation	4.26 ± 0.59	1–5	−0.71	0.03	0.78	0.12		
	Role acquisition	4.38 ± 0.63	1–5	−1.22	1.45	0.69	0.13		
	Self-provided	3.74 ± 0.65	1–5	−0.07	−0.19	0.74	0.14		
Health-related quality of life	Overall	3.45 ± 0.77	1–5	−0.51	0.79	0.68		0.95	0.79
	Physical	3.37 ± 0.63	1–5	−0.36	0.30	0.75	0.09		
	Psychological	3.60 ± 0.57	1–5	−0.27	−0.14	0.87	0.08		
	Social	3.58 ± 0.61	1–5	−0.10	−0.01	0.79	0.09		
	Environmental	3.67 ± 0.59	1–5	−0.20	−0.24	0.80	0.08		

AVE = Average variance extracted; CR = Construct reliability; M = Mean; SD = Standard deviation; SE = Standard error.

**Table 3:** Model fit (n = 200).

Fit Index	$\chi^{2}$ (*p*)	df	$\chi^{2}$/df	SRMR	RMSEA	TLI	CFI
Criteria	*p* > 0.05		≤3	≤0.05	≤0.08	≥0.9	≥0.9
Hypothetical model	456.81 (<0.001)	185	2.47	0.02	0.08	0.83	0.87
Modified model	395.70 (<0.001)	185	2.20	0.02	0.07	0.86	0.90

CFI = Comparative fit index; RMSEA = Root mean square error of approximation; SRMR = Standardized root mean square residual; TLI = Tucker–Lewis index.

**Table 4:** Parameter estimates result of the modified model (n = 200).

Endogenous Variables	Exogenous Variables	SC	SE	CR	*p*	SMC	Adoption
Number of diseases	←Age	0.44	0.05	7.32	<0.001	0.27	**Accept**
	←Pregnancy week	−0.31	0.03	−5.13	<0.001		**Accept**
	←Planned pregnancy	0.03	0.10	0.61	0.539		Reject
	←Social support	−0.02	0.12	−0.29	0.770		Reject
	←Marital intimacy	−0.00	0.16	−0.07	0.939		Reject
Antenatal depression	←Age	−0.07	0.04	−0.99	0.318	0.26	Reject
	←Pregnancy week	−0.25	0.02	−3.81	<0.001		**Accept**
	←Planned pregnancy	−0.10	0.07	−1.71	0.087		Reject
	←Social support	−0.29	0.08	−3.28	0.001		**Accept**
	←Marital intimacy	−0.18	0.11	−2.01	0.044		**Accept**
	←Number of disease	0.02	0.05	0.30	0.759		Reject
Self-care behavior	←Age	0.07	0.03	1.15	0.249	0.29	Reject
	←Pregnancy week	0.12	0.02	1.83	0.067		Reject
	←Planned pregnancy	0.20	0.05	3.34	<0.001		**Accept**
	←Social support	0.13	0.06	1.54	0.123		Reject
	←Marital intimacy	0.33	0.08	3.58	<0.001		**Accept**
	←Number of disease	−0.09	0.03	−1.36	0.173		Reject
	←Antenatal depression	−0.05	0.05	−0.72	0.466		Reject
Fetal attachment	←Age	0.12	0.04	1.78	0.074	0.39	Reject
	←Pregnancy week	0.07	0.02	1.01	0.312		Reject
	←Planned pregnancy	0.20	0.05	3.34	<0.001		**Accept**
	←Social support	0.02	0.08	0.24	0.810		Reject
	←Marital intimacy	0.32	0.12	3.12	0.002		**Accept**
	←Number of disease	−0.02	0.04	−0.27	0.787		Reject
	←Antenatal depression	−0.05	0.06	−0.77	0.437		Reject
Perceived health condition	←Age	0.14	0.06	1.95	0.051	0.46	Reject
	←Pregnancy week	0.12	0.04	1.62	0.103		Reject
	←Planned pregnancy	−0.06	0.10	−0.87	0.381		Reject
	←Social support	0.07	0.12	0.74	0.455		Reject
	←Marital intimacy	0.01	0.18	0.13	0.890		Reject
	←Number of disease	−0.14	0.07	−1.84	0.065		Reject
	←Antenatal depression	−0.10	0.10	−4.14	0.159		Reject
	←Self -care behavior	0.17	0.15	2.11	0.035		**Accept**
	←Fetal attachment	0.02	0.13	0.23	0.811		Reject
Health-related quality of life	←Age	0.05	0.03	1.11	0.265	0.73	Reject
	←Pregnancy week	0.05	0.02	0.97	0.328		Reject
	←Planned pregnancy	0.04	0.05	0.85	0.391		Reject
	←Social support	0.12	0.06	1.90	0.057		Reject
	←Marital intimacy	0.26	0.10	3.30	<0.001		**Accept**
	←Number of disease	−0.09	0.03	−1.75	0.080		Reject
	←Antenatal depression	−0.27	0.05	−4.84	<0.001		**Accept**
	←Self-care behavior	0.12	0.08	2.09	0.037		**Accept**
	←Fetal attachment	0.25	0.07	3.63	<0.001		**Accept**
	←Perceived health condition	0.14	0.03	2.79	0.005		**Accept**

CR = Critical ratio; SC = Standardized coefficient; SE = Standard error. Note: Arrows (←) indicate the direction of influence from exogenous variables to endogenous variables. Bold indicates statistically significant values (*p* < 0.05).

**Table 5:** Direct effects, indirect effects, and total effects in modified model (n = 200).

Endogenous Variables	Exogenous Variables	Direct Effect	Indirect Effect	Total Effect
		β (*p*)	β (*p*)	β (*p*)
Number of diseases	←Age	0.44 (<0.001)		0.44 (<0.001)
	←Pregnancy week	−0.31 (<0.001)		−0.31 (<0.001)
	←Planned pregnancy	0.03 (0.539)		0.03 (0.539)
	←Social support	−0.02 (0.770)		−0.02 (0.770)
	←Marital intimacy	−0.00 (0.939)		−0.00 (0.939)
Antenatal depression	←Age	−0.07 (0.318)	0.01 (0.748)	−0.06 (0.165)
	←Pregnancy week	−0.25 (<0.001)	−0.00 (0.730)	−0.26 (0.011)
	←Planned pregnancy	−0.10 (0.087)	0.00 (0.557)	−0.10 (0.089)
	←Social support	−0.29 (0.001)	−0.00 (0.620)	−0.29 (0.010)
	←Marital intimacy	−0.18 (0.044)	0.00 (0.888)	−0.18 (0.103)
	←Number of disease	0.02 (0.759)		0.02 (0.759)
Self-care behavior	←Age	0.07 (0.249)	−0.04 (0.220)	0.03 (0.498)
	←Pregnancy week	0.12 (0.067)	0.04 (0.118)	0.16 (0.011)
	←Planned pregnancy	0.20 (<0.001)	0.00 (0.786)	0.20 (0.013)
	←Social support	0.13 (0.123)	0.01 (0.327)	0.15 (0.177)
	←Marital intimacy	0.33 (<0.001)	0.01 (0.437)	0.34 (0.008)
	←Number of disease	−0.09 (0.173)	−0.00 (0.559)	−0.09 (0.191)
	←Antenatal depression	−0.05 (0.466)		−0.05 (0.466)
Fetal attachment	←Age	0.12 (0.074)	0.00 (0.825)	0.13 (0.087)
	←Pregnancy week	0.07 (0.312)	0.07 (0.042)	0.14 (0.067)
	←Planned pregnancy		0.07 (0.005)	0.07 (0.005)
	←Social support	0.02 (0.810)	0.07 (0.088)	0.09 (0.538)
	←Marital intimacy	0.32 (0.002)	0.12 (0.003)	0.44 (0.007)
	←Number of disease	−0.02 (0.787)	−0.03 (0.196)	−0.05 (0.191)
	←Antenatal depression	−0.05 (0.437)	−0.01 (0.329)	−0.07 (0.395)
Perceived health condition	←Age	0.14 (0.051)	−0.04 (0.179)	0.10 (0.319)
	←Pregnancy week	0.12 (0.103)	0.10 (0.005)	0.24 (0.011)
	←Planned pregnancy	−0.06 (0.381)	0.04 (0.055)	0.11 (0.834)
	←Social support	0.07 (0.455)	0.06 (0.072)	0.27 (0.266)
	←Marital intimacy	0.01 (0.890)	0.09 (0.096)	0.10 (0.407)
	←Number of disease	−0.14 (0.065)	−0.02 (0.145)	−0.14 (0.069)
	←Antenatal depression	−0.10 (0.159)	−0.01 (0.323)	−0.11 (0.112)
	←Self -care behavior	0.17 (0.035)	0.00 (0.780)	0.18 (0.054)
	←Fetal attachment	0.02 (0.811)		0.02 (0.811)
Health-related quality of life	←Age	0.05 (0.265)	0.02 (0.585)	0.08 (0.215)
	←Pregnancy week	0.05 (0.328)	0.19 (0.008)	0.24 (0.021)
	←Planned pregnancy	0.04 (0.391)	0.06 (0.010)	0.11 (0.123)
	←Social support	0.12 (0.057)	0.14 (0.017)	0.27 (0.044)
	←Marital intimacy	0.26 (<0.001)	0.22 (0.006)	0.48 (0.005)
	←Number of disease	−0.09 (0.080)	−0.05 (0.189)	−0.14 (0.022)
	←Antenatal depression	−0.27 (<0.001)	−0.04 (0.148)	−0.31 (0.007)
	←Self -care behavior	0.12 (0.037)	0.11 (0.007)	0.23 (0.011)
	←Fetal attachment	0.25 (<0.001)	0.00 (0.897)	0.25 (0.012)
	←Perceived health condition	0.14 (0.005)		0.14 (0.005)

Note: Arrows (←) indicate the direction of influence from exogenous variables to endogenous variables.

## Data Availability

The data presented in this study are available on request from the corresponding author. The data are not publicly available due to privacy concerns.

## Abbreviations

The following abbreviations are used in this manuscript:

HRQoL	Health-related quality of life
MFICU	Maternal-fetal intensive care unit
